# Photothermal inactivation of universal viral particles by localized surface plasmon resonance mediated heating filter membrane

**DOI:** 10.1038/s41598-022-05738-2

**Published:** 2022-02-02

**Authors:** Seunghwan Yoo, Sun-Woo Yoon, Woo-Nam Jung, Moon Hyun Chung, Hyunjun Kim, Hagkeun Jeong, Kyung-Hwa Yoo

**Affiliations:** 1 Energy ICT Convergence Research Department, Energy Efficiency Research Division, Korea Institute of Energy Research, 152 Gajeong-ro, Yuseong-gu, Daejeon, 34129 Republic of Korea; 2 Department of Physics, Yonsei University, 50 Yonsei-ro, Seodaemun-gu, Seoul, 03722 Republic of Korea; 3 Biotechnology Research Center, Korea Research Institute of Bioscience and Biotechnology, 125 Gwahak-ro, Yuseong-gu, Daejeon, 34141 Republic of Korea; 4 University of Science and Technology, 217 Gajeong-ro, Yuseong-gu, Daejeon, 34114 Republic of Korea; 5 Advanced Combustion Power Lab., Energy Efficiency Research Division, Korea Institute of Energy Research, 152, Gajeong-ro, Yuseong-gu, Daejeon, 34129 Republic of Korea; 6 Department of Mechnical Engineering, Korea Advanced Institute of Science and Technology, 291 Daehak-ro, Yuseong-gu, Deajeon, 34141 Republic of Korea; 7 Energy Efficiency Research Division, Korea Institute of Energy Research, 152, Gajeong-ro, Yuseong-gu, Daejeon, 34129 Republic of Korea

**Keywords:** Nanoparticles, Nanophotonics and plasmonics, Influenza virus, SARS-CoV-2, Natural hazards

## Abstract

This study introduces localized surface plasmon resonance (L-SPR) mediated heating filter membrane (HFM) for inactivating universal viral particles by using the photothermal effect of plasmonic metal nanoparticles (NPs). Plasmonic metal NPs were coated onto filter membrane via a conventional spray-coating method. The surface temperature of the HFM could be controlled to approximately 40–60 °C at room temperature, owing to the photothermal effect of the gold (Au) NPs coated on them, under irradiation by visible light-emitting diodes. Due to the photothermal effect of the HFMs, the virus titer of H1Npdm09 was reduced by > 99.9%, the full inactivation time being < 10 min, confirming the 50% tissue culture infective dose (TCID_50_) assay. Crystal violet staining showed that the infectious samples with photothermal inactivation lost their infectivity against Mardin-Darby Canine Kidney cells. Moreover, photothermal inactivation could also be applied to reduce the infectivity of SARS-CoV-2, showing reduction rate of 99%. We used quantitative reverse transcription polymerase chain reaction (qRT-PCR) techniques to confirm the existence of viral genes on the surface of the HFM. The results of the TCID_50_ assay, crystal violet staining method, and qRT-PCR showed that the effective and immediate reduction in viral infectivity possibly originated from the denaturation or deformation of membrane proteins and components. This study provides a new, simple, and effective method to inactivate viral infectivity, leading to its potential application in various fields of indoor air quality control and medical science.

## Introduction

After the first patient infected with the coronavirus disease 2019 (COVID-19) was identified in December 2019, the rapid spread of the tiny but very active corona-shaped viruses were noticed around the globe, resulting in explosive infectivity^1^. To contain the COVID-19 pandemic, researchers in wide-ranging fields including medicine, pharmacy, and nanotechnology began exploring effective virus inactivation processes that might allow the world to return to normalcy^2,3^. As a representative example, pharmaceutical companies accelerated the development of vaccines and medicines^4–6^. Moreover, various detection methods were developed, such as nanodevice-based biosensing^7–10^, quantitative reverse transcription polymerase chain reaction (qRT-PCR)^11–13^, and immunoassays^14^. Since viral transmission via small airborne particles, referred to as aerosols, was noted as a significant cause of the worldwide pandemic, blocking the pathway of viral transmission became increasingly important^15–17^.

According to the hierarchy of traditional infection control adapted from the US Centers for Disease Control and Prevention, the stage of “Engineering Controls” emphasizes reducing the potential airborne transmission of viral particles through continuous ventilation, cleaning of indoor air and contaminated areas, the use of disinfection devices, and reducing the number of people in gatherings^18^. In particular, airborne viral particles circulate indoors, eventually transmitting to other spaces through the ventilation system^19,20^. However, tiny particles such as fine dust, airborne bacteria, and viruses can be captured during ventilation using high-efficiency particulate air (HEPA) filters^21–23^. Nevertheless, based on the final stage of the hierarchy of traditional infection control, the most effective infection control method is to physically eliminate or inactivate pathogens. Moreover, it is important to prevent the growth of pathogens on the filter membrane itself.

For physical elimination or inactivation of pathogens, several methods are available, including environmental conditions, steam sterilization, alcohol washing, bleach washing, ultraviolet (UV) irradiation, and dry heat treatment^24–30^. Among these methods, UV irradiation can be adopted for ventilation and filtering systems. However, a high dose of UV irradiation causes significant degradation of various materials, including filter fibers. Moreover, UV light does not permeate crevices—thus, viruses within crevices may not be completely killed by UV irradiation^31,32^.

Generally, for viral infection in mammalian cells, viruses first touch the host cell membrane^33^. For example, the entry of the influenza A virus—A/California/04/2009(H1N1pdm09)—begins with the attachment of its membrane protein, hemagglutinin (HA), to the cell surface receptor sialic acid (SA) embedded in the lipid bilayer of the host cell^34^. If the virus is exposed to heat, the heat induces structural deformation of the virus membrane protein or its components—that is, fusion or attachment of the HA protein to SA is prevented, and the infection process ceases immediately (Fig. S1).

In this study, we developed a heating filter membrane (HFM) coated with plasmonic gold nanoparticles (Au NPs) and investigated whether the virus on the surface of the HFM could be inactivated by visible light illumination. When the HFM was exposed to visible light for 10 min, the temperature increased up to ∼ 60 °C and the viral infectivity—measured using the TCID_50_ assay and crystal violet staining method—decreased by ∼ 99.9%. Furthermore, qRT-PCR was conducted to confirm the existence of viral particles on the surface of the HFM, remaining in a non-infectious state. The results showed that viral particles—especially the H1N1pdm09 virus in this study—could be effectively and physically inactivated. We conducted similar experiments with SARS-CoV-2 viruses and found that it could also be inactivated via photothermal effects. These results demonstrated that the photothermal inactivation method could be an effective approach for improving inactivation methods of general airborne viruses captured by filter materials and pre-treatment of virus samples derived from infected patients.

## Results and discussion

### Simulation results of photothermal effect

**Figure 1:**
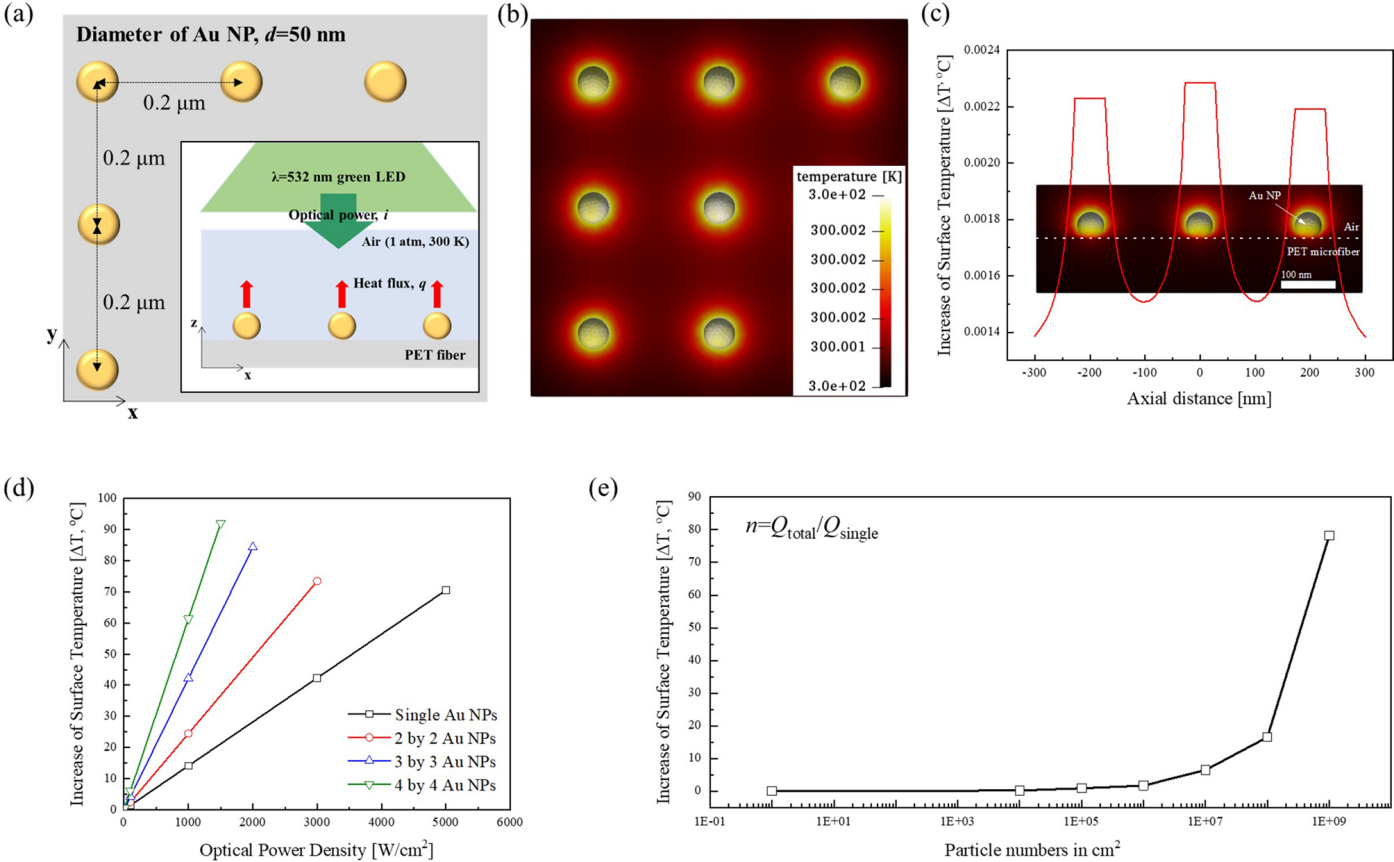
Simulation of the photothermal effect generated by Au NPs on a PET microfiber supporting layer. (**a**) Configuration of Au NPs arrays on the PET microfiber and LED light irradiation. The inset shows a side view of the Au NPs array. (**b**, **c**) Top view and side view of temperature distribution by the photothermal effect of 3 × 3 Au NPs array under an optical power density of 100 mW/cm^2^. (**d**) Variation of surface temperature with respect to the size of Au NPs arrays under different optical power densities. (**e**) Simplified estimation for surface temperature versus particle numbers per cm^2^ under an optical power density of 100 mW/cm^2^.

For the simulation of the photothermal effect and temperature distribution generated by the HFM coated with Au NPs, we set up the model as shown in Fig. 1a. We assumed the size (diameter) of the Au NPs to be approximately 50 nm and the distance between the Au NPs to be 0.2 μm from each other, the Au NPs contact being point-like on the surface of the HFM. The surrounding materials were air (density: 1.225 kg/m^3^, specific heat: 1006.43 J/kg·K, thermal conductivity: 0.0242 W/m·K) and PET microfiber (density: 1380 kg/m^3^, specific heat: 1000 J/kg·K, thermal conductivity: 0.2 W/m·K). The simulation variables were light intensity, number of particles on the filter material, and the size of the Au NP.

Under visible light illumination, a single Au NP can generate local heat power, the heat source arisings from the Joule effect^35,36^ :

1 $$ Q_{single} = C_{abs} \cdot I $$

where Q_single_ is the local heat generated by a single Au NP, C_abs_ and I are the absorption cross section of a single Au NP and the incident optical power density, respectively. Moreover, the temperature change or increase around the Au NP array is due to the total number of Au NPs—the so-called ensemble effect—which can contribute to the total heat source (Q_total_). With a certain number of Au NPs, the heat generation referred to as the specific absorption rate (SAR, W/m^3^) is the sum of individual heat generation by all Au NPs, which can be defined as follows^37,38^:

2 $$ SAR = Q_{total} = \sum Q_{single} = N \cdot C_{abs} \cdot I $$

Thus, in the absence of phase change and heat convection, the heat diffusion equation can be expresses as follows:

3 $$ \rho c\frac{dT}{{dt}} = \kappa \nabla^{2} T + Q_{total} = \kappa \nabla^{2} T + N \cdot C_{abs} \cdot I $$

Based on Eq. (1–3), Fig. 1b, c show the top and side views, respectively, of the temperature distribution by the photothermal effect of a 3 × 3 Au NP array. A single Au NP can generate L-SPR-driven heat energy and elevate the temperature up to approximately 2 mK under an optical power density of 100 mW/cm^2^ (Fig. S2). Moreover, the temperature at the center of the Au NP array was slightly higher than that of the neighboring Au NPs owing to the ensemble effect^38^.

We also simulated the increase in the surface temperature of the HFM with respect to the number of Au NP arrays and the incident optical power density, as shown in Fig. 1d. All arrays of Au NPs can generate heat and increase the surface temperature of the HFM linearly with the number of Au NP arrays and the increase in the incident optical power density. Finally, to inactivate the viral particles, we estimated the number of Au NPs per cm^2^ needed to elevate the surface temperature to 60 °C at room temperature (RT, 20–25 °C) under an optical power density of 100 mW/cm^2^, as shown in Fig. 1e. As a result, approximately 10^8^–10^9^ Au NP particles per cm^2^ are needed to raise the surface temperature of the HFM to inactivate the viral particles.

### Characterization of the fabricated HFM

**Figure 2:**
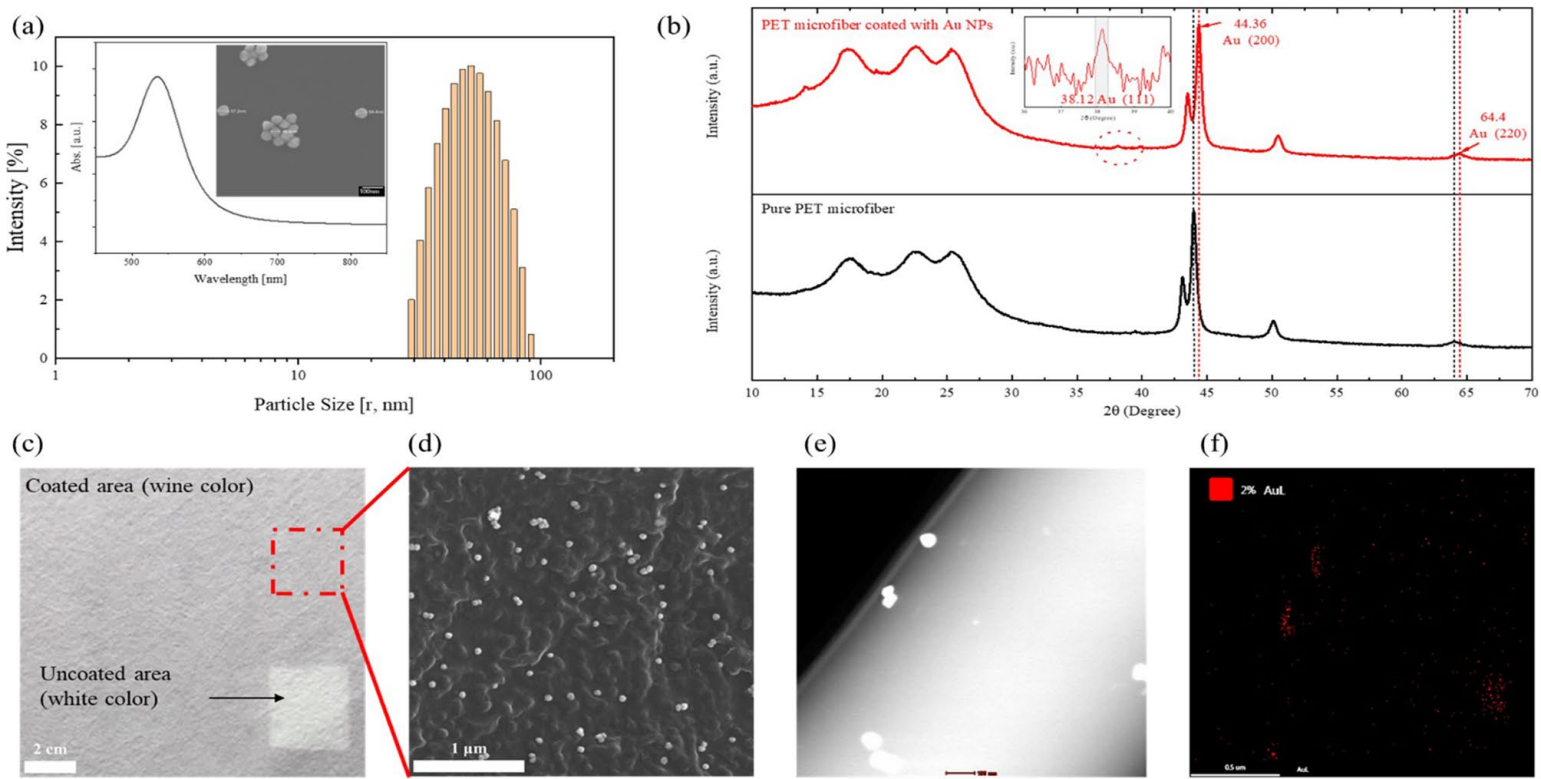
Characterization of the as-fabricated HFM. (**a**) Particle size, UV–Vis absorbance, and SEM image of the synthesized Au NPs. (**b**) XRD patterns of the PET microfiber with and without Au NPs. (**c**) Optical image of the HFM. (**d**, **e**) SEM and TEM image of the HFM coated with Au NPs. (**f**) TEM-EDS image of Au NPs on the PET microfiber.

Based on the simulation results of the photothermal effect, we first prepared the HFM using a commercial HEPA filter material, conventionally spray-coated with plasmonic Au NPs. As shown in Fig. 2a, the average size and absorption peak of the as-synthesized Au NPs were approximately 50 nm in diameter and 532 nm in wavelength, respectively. We defined the effective particle number of Au NPs on the surface of the HFM to be approximately 5.0 × 10^8^ per cm^2^ for photothermal inactivation and calculated the optical density (OD) of the Au NPs suspension based on the method suggested by W. Haiss et al. in consideration of the amount of waste during the spray-coating process^39^. The final concentration and volume of spray-coating solution were set at 0.5 OD and 1 mL/cm^2^ of Au NPs solution, respectively.

The XRD patterns of the fabricated HFM—especially the PET supporting layer—coated and uncoated (pure filter membrane, PFM) with the Au NPs are shown in Fig. 2b. Compared with the peak of the PFM, the HFM exhibits an XRD peak of Au (111) at 38.12° of 2θ. In addition, in the case of the HFM, the peaks around 44 and 64 shifted slightly to 44.36 for Au (200) and 64.40 for Au (220), respectively^40^. This peak shift of the HFM to a higher angle may originate from the Au NPs on the surface of the HFM. We also investigated the FTIR spectrum, which showed that there was no chemical change in the HFM (Fig. S3).

Figure 2c–f shows the optical, SEM, and TEM images of the HFM coated with Au NPs on the PET layer. The coated and uncoated areas displayed different colors that is, red-wine and white, respectively. Moreover, as seen in the SEM, TEM and TEM-EDS images of Fig. 2c–f, the Au NPs were uniformly spray-coated on the surface of the HFM.

### Photothermal effect of the HFM

**Figure 3:**
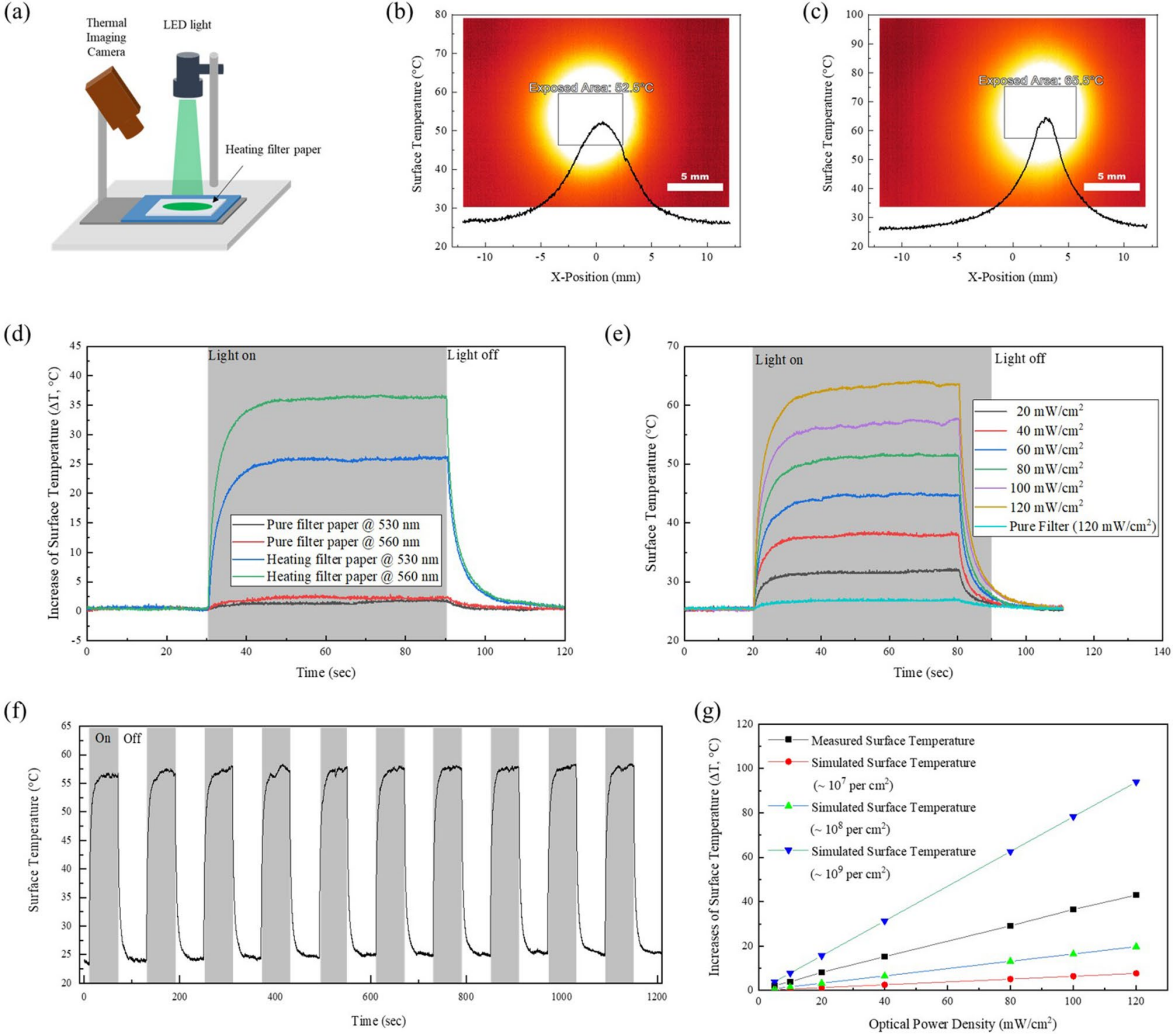
Evaluations of the photothermal effect of HFM. (**a**) Measurement set-up of the photothermal effect of the HFM under visible light irradiation (530 nm and 560 nm LED light sources). (**b**, **c**) TIC images and temperature profiles of the HFM under 530 nm and 560 nm LED light irradiation at an optical power density of 100 mW/cm^2^, respectively, at room temperature. The scale bar is 5 mm. (**d**) The measured surface temperature changes of HFM and PFM with respect to the wavelength of incident light, the wavelengths of incident light (530 nm and 560 nm). (**e**) The measured surface temperature of the HFM with respect to an incident optical power density of 560 nm LED irradiation at RT. For the PFM, the surface temperature was measured at an optical power density of 120 mW/cm^2^. (**f**) Repeatable heating plots of the HFM under 560 nm LED irradiation at an optical power density of 100 mW/cm^2^ at room temperature. (**g**) Comparative plots of measured and simulated surface temperatures with respect to the optical power density of the incident light and the volume of Au NPs coated on the HFM.

The surface temperature of the HFM, which originated from the photothermal effect of the plasmonic Au NPs, were investigated using thermal imaging techniques (TIC). The measurement setup for the photothermal effect of the HFM is illustrated schematically in the inset of Fig. 3a. Under visible LED light irradiation, we first measured the surface temperatures of the HFM with wavelengths of 530 and 560 nm LED lights using TIC.

As shown in Fig. 3b, c, the resultant surface temperatures after 10 s of irradiation at 100 mW/cm^2^ at RT were approximately 52 and 65 °C under 530 and 560 nm LED light irradiation, respectively (see video clips in supplementary materials). Compared with the temperature of the surrounding surface, the surface temperature irradiated by the 530 and 560 nm LED lights increased by 25 and 35 °C, respectively, over and above that of the normal surface, the PFM showing no increase in surface temperature, as shown in Fig. 3d. Although the absorbance of Au NPs dispersed in deionized (DI) water showed a maximum peak at 532 nm, the HFM showed a higher surface temperature under the 560 nm LED light than the 530 nm LED light. This red-shift in the absorption wavelength, which could generate the photothermal effect, was caused primarily by the change in the dielectric constant of the surrounding medium, such as the DI water, to PET microfibers, and by changes in the diameter of Au NPs due to aggregation during evaporation of the solvent^41^.

Figure 3e shows the measured surface temperatures of the HFM with respect to the incident optical power density of 560 nm LED light irradiation, the surface temperatures of the HFM increasing at a speed of 0.35 °C/mW (Fig. S4). Moreover, the HFM did not show dramatic surface temperature changes even though irradiated by the higher optical power density (120 mW/cm^2^) of the 560 nm LED, as shown in Fig. 3c, which led to an increase in the surface temperature of the HFM originating only from the photothermal effect of Au NPs under visible light irradiation (linear with respect to the absorbed optical power density). Thereafter, the photothermal stability of the HFM was tested for at least 100 consecutive cycles of surface temperature profiles under 560 nm LED light irradiation with an optical power density of 100 mW/cm^2^, as shown in Figs. 3f and S5. And, we also monitored the surface temperature fluctuation under continuous exposure of 560 nm LED light irradiation for 1 h (Fig. S6). This result shows that the HFM had high photothermal stability and repeatability, which is considered suitable for repeated experiments.

Figure 3g shows the measured and simulated surface temperatures of the HFM. Comparing the measured surface temperature of the HFM with the simulated results, the Au NPs were uniformly coated on the surface of the HFM, the number of Au NPs on the surface being expected to be between 10^8^ and 10^9^ per cm^2^ (as mentioned in the fabrication and simulation processes of the HFM). These surface temperatures were sufficient for irreversibly injuring biological specimens, including viral particles, leading to a dramatic activation of cell death above 48 °C and instantaneous and irreversible protein denaturation above 60 °C^42^.

### Evaluation of photothermal inactivation of the H1N1pdm09 virus

**Figure 4:**
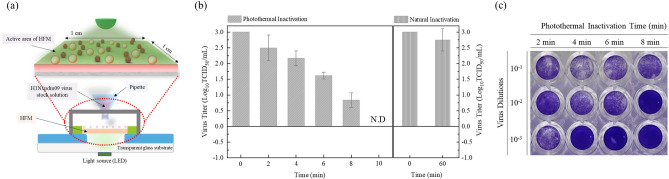
Assessment of the titer of the H1N1pdm09 virus with respect to the PTI time. (**a**) Schematic view of the PTI kit. (**b**) Result of the TCID_50_ assay with respect to the PTI time and natural inactivation. (**c**) Photographs of the crystal violet staining of the MDCK cells with respect to the PTI time.

For the photothermal inactivation (PTI) of the H1N1pdm09 virus, the HFM was placed in the inactivation kit and the reference stock of the H1N1pdm09 virus was loaded onto its surface, as shown in Fig. 4a. After the virus stock solutions were loaded by using the pipette, the PTI was conducted with a visible 560 nm LED light. The optical power density of the incident LED light source (wavelength: 560 nm) and the irradiated active area of the filter membrane were fixed at 100 mW/cm^2^ and 1 × 1 cm^2^, respectively, in all the PTI experiments, all of which were sterilized by UV-C irradiation for 20 min before use to prevent contamination.

We first investigated the inactivation time of the H1N1pdm09 virus (3log_10_TCID_50_/mL or 3-log) under the photothermal effect, as shown in Fig. 4b. With respect to the PTI time, the titers of the H1N1pdm09 virus of 3-log were 2.5 ± 0.4, 2.1 ± 0.2, 1.6 ± 0.1, 0.8 ± 0.2, and 0 under 2, 4, 6, 8, and 10 min of PTI time, respectively, suggesting that the infectivity of the H1N1pdm09 virus was fully inactivated within 10 min. In addition, the H1N1pdm09 virus had its own infectivity without photothermal inactivation after 1 h. Figure 4c shows photographs of the crystal violet staining and the CPE of the MDCK cells with respect to the PTI time, and the stained MDCK cells can be seen based on the infectivity reduction rate. To confirm the effect of the duration of PTI, we sequentially conducted PTI under the same conditions by varying the irradiation time from 10 to 40 min. None of the MDCK cells in the well plate were infected with the H1N1pdm09 virus, showing that PTI for just 10 min was sufficient to inactivate the H1N1pdm09 virus (Fig. S7).

Based on the simulation results of an earlier study, a 3-log reduction in the concentration of the virus—which normally represents a 99.9% reduction—could be achieved in approximately 3 min under exposure to a temperature of 70 °C ^43^. In addition, Tulandhar et al.^44^ reported that a 3-log infectivity reduction of the H1N1 virus could be achieved after 30 min when the H1N1 virus suspended in DMEM was treated at 56 °C. Generally, inactivation methods such as UV-C and heat treatment recommend that the inactivation time should be > 15 min, making PTI a simple and sufficient inactivation method for virus infectivity.

**Figure 5:**
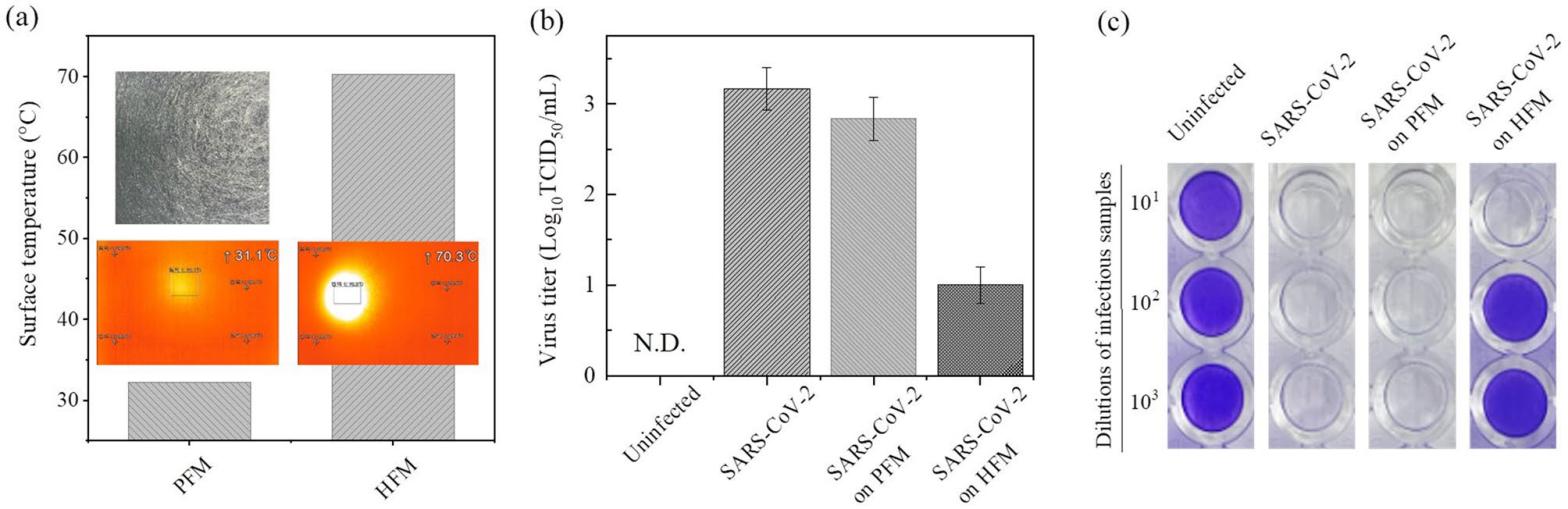
Test for photothermal inactivation of the SARS-CoV-2 virus using the s-HFM. (**a**) Surface temperature of the s-HFM under visible light illumination (100 mW/cm^2^), (**b**) Result of the TCID_50_ assay, and (**c**) Photograph of the crystal violet staining of the MDCK cells with and without PTI.

Subsequently, we also performed the photothermal inactivation of SARS-CoV-2 virus with the HFM. As shown in Fig. S8, the plaque assay showed that the photothermal inactivation of SARS-CoV-2 virus was less effective than that of H1N1pdm09 virus. In the case of SARS-CoV-2 virus, when the concentration of SARS-CoV-2 virus is 4-log or more, the photothermal inactivation of SARS-CoV-2 virus dropped sharply. This shows that the SARS-CoV-2 virus is more contagious or infective than H1N1pdm09 virus, and thus the photothermal inactivation is lower. Therefore, we newly introduced and fabricated an HFM with Au/TiO_2_ deposited using a sputtering evaporator (s-HFM, Fig. S9), showing higher surface temperature than the HFM. The thicknesses of the Au and TiO_2_ layers were 10 and 200 nm, respectively. Figure 5a shows the surface temperature of the PFM and s-HFM under 560 nm LED light irradiation (optical power density = 100 mW/cm^2^). The surface temperatures of the PFM and s- HFM increased approximately up to 31 and 70 °C, respectively at RT. We tested the PTI of the SARS-CoV-2 virus (BetaCoV/South Korea/2020, 3-log) using the PFM and s-HFM (Fig. 5)b, c. Upon visible light illumination, the virus titer of the SARS-CoV-2 virus on the s- HFM decreased from 3-log to 1-log in comparison with the virus stock of original SARS-CoV-2 virus, leading to a 99% reduction in viral infectivity, while the virus titer on the PFM decreased only slightly. These results show that PTI could be applied to several corona-shaped viral particles, including the H1N1pdm09 and SARS-CoV-2 viruses.

**Figure 6:**
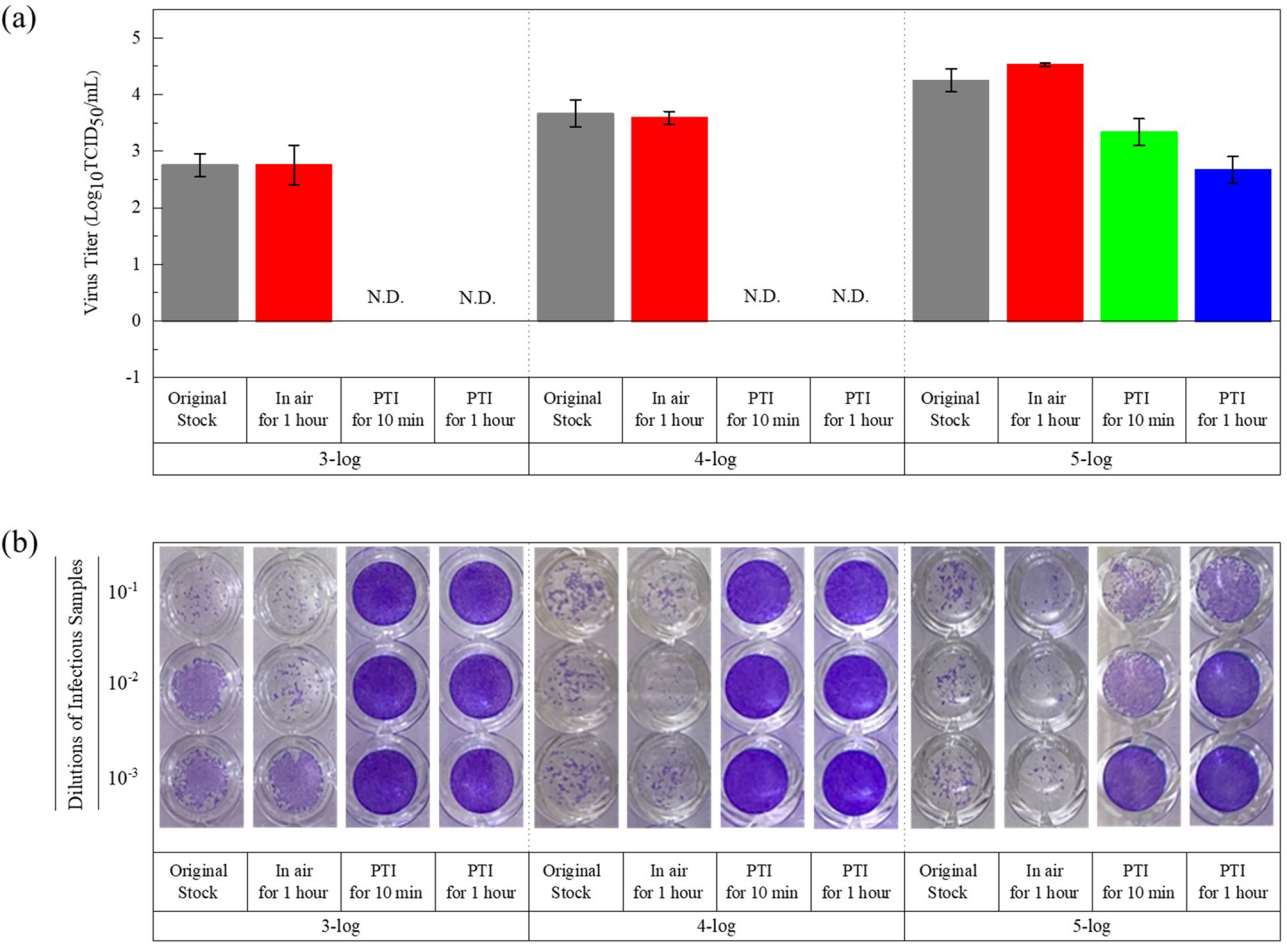
Assessment of the titer of the H1N1pdm09 virus with respect to the viral doses (or concentrations) and inactivation times. (**a**) Results of the TCID_50_ assay, and (**b**) Photographs of the crystal violet staining of the MDCK cells with respect to the dose of the virus stock, the exposure time of PTI.

Thereafter, we prepared three doses of the reference stocks of the H1N1pdm09 virus (low dose: 3-log; medium dose: 4log_10_TCID_50_/mL or 4-log; high dose: 5log_10_TCID_50_/mL or 5-log). Equal volumes (100 μL) of the H1N1pdm09 virus were coated onto the surface of the PFM without PTI and the HFM with PTI. Figure 6a shows the assessed titer of the H1N1pdm09 virus subjected to PTI for 10 min and 1 h. All infectious samples of 3-log, 4-log, and 5-log original reference stocks showed their own infectivity, and the infectious samples of the PFM remained in the air for 1 h, also maintaining high viral infectivity. By contrast, the infectious samples derived from the HFM with doses of 3-log and 4-log were fully inactivated under PTI for both 10 min and 1 h. Figure 6b shows photographs of the crystal violet staining and the CPE of the MDCK cells with respect to the dose of virus stock and exposure time of PTI. Without PTI, all infectious samples remaining on the surface of the PFM in the air were the most viable after 1 h—they could infect the MDCK cells and induce CPE in them. With PTI for 10 min and 1 h, more than 99.9% of the H1N1pdm09 virus was removed or their infectivity was lost. Consequently, no CPE was observed in both the 3-log and 4-log cases.

However, although dramatic reduction of the viral infectivity was observed at 3-log and 4-log concentrations of the virus stock, the virus titer of the 5-log case decreased logarithmically from 5.0-log to 3.3-log and from 5.0-log to 2.67-log after 10 min and 1 h of PTI, respectively. When the dose of virus stock was 5-log, the CPE of the MDCK cells was also observed, as shown in 6b. These logarithmic reduction rates show that they decreased from 10^5^ to 10^3.3^ and from 10^5^ to 10^2.67^, leading to reduction rates of 97% and 99.7%, respectively. This result may originate from the absolute population of the live viruses in the infectious samples. Furthermore, the PTI kit or the HFM might have had limited inactivation capability due to the restricted active area of 1 × 1 cm^2^.

### The existence of the H1N1pdm09 virus on the HFM

To confirm the existence of the H1N1pdm09 virus on the surface of the HFM, we performed quantitative reverse transcription polymerase chain reaction (qRT-PCR) assay, which is normally used for the detection of all types of viruses, especially the M-gene for influenza A viruses. Here, we used the sequence of 5′-FAM-TCA GGC CTC AAA GCC GA-TAMRA-3′ as a double-labelled probe to detect the M-gene of the H1N1pdm09 virus (Table 1). We prepared infectious samples derived from the PFM and HFM, including the H1N1pdm09 virus reference stock of 7-log as a high-dose sample and 3-log as a normal dose sample.

**Table 1:** PCR primer and hydrolysis probe sequences for the qRT-PCR of the H1N1pdm09 virus. The probe was designed for all-type-A influenza virus M-genes^58^.

Prime/probe	Sequence (5′-3′)
Forward primer (M + 25)	5′-AGA TGA GTC TTC TAA CCG AGG TCG-3′
Probe (M + 64)	5′-FAM-TCA GGC CCC CTC AAA GCC GA-TAMRA-3′
Reverse primer (M-124)	5′-TGA AAA AAC ATC TTC AAG TCT CTG-3′

**Figure 7:**
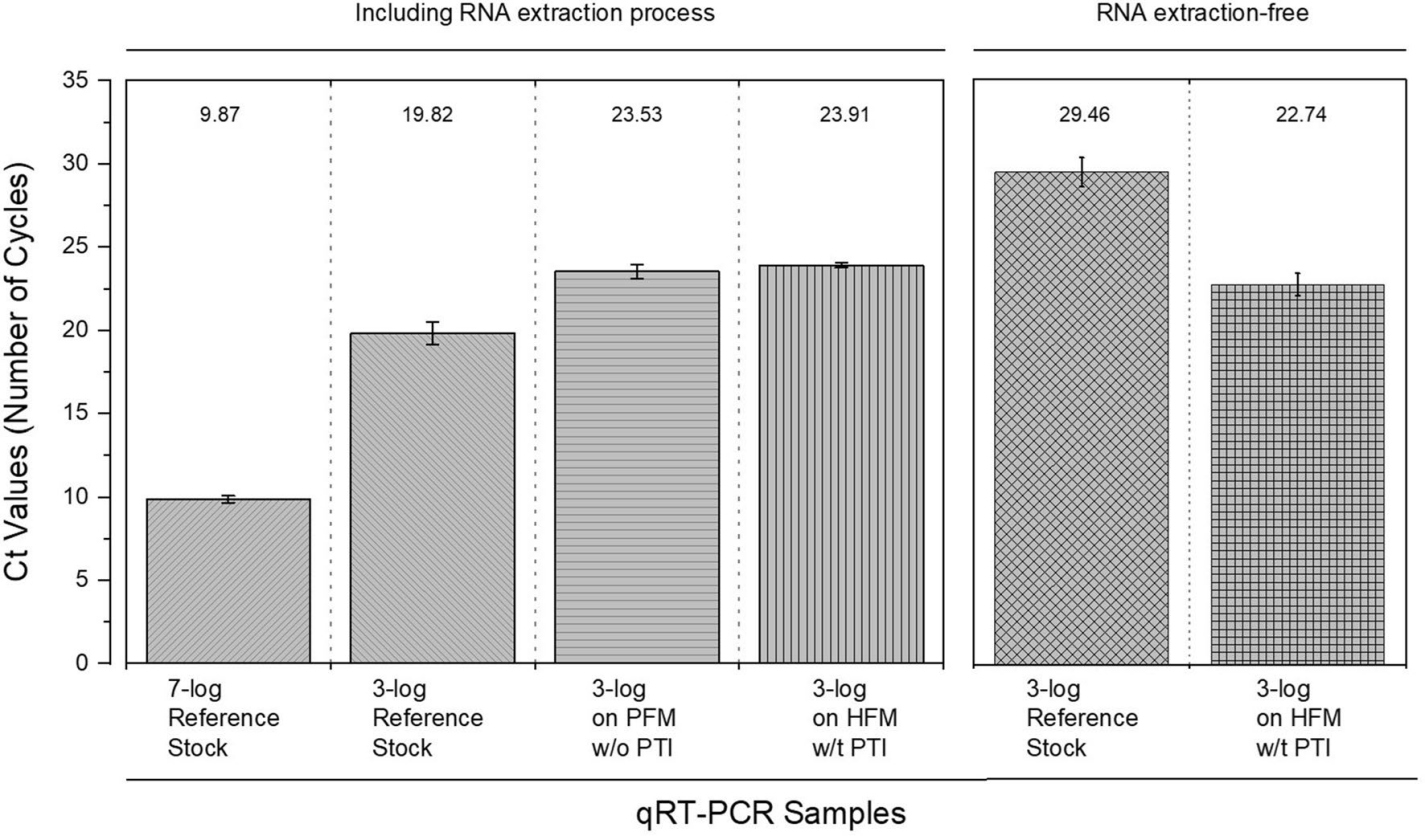
Comparison of the cyclic threshold (Ct) values of each infectious sample for detecting the M-gene of the H1N1 pdm09 virus.

Figure 7 shows the cycle threshold (Ct) values for each infectious sample. The average Ct values of each sample were 9.87 for 7-log H1N1pdm09, 19.82 for 3-log, 23.53 for the PFM without PTI, and 23.91 for the HFM with PTI. These results show that the M-gene of the H1N1pdm09 virus was seen in all infectious samples. In particular, the Ct values of both the PFM without PTI and HFM with PTI were almost the same, implying that the same concentration (or dose) of the H1N1pdm09 virus existed on both the PFM and HFM surfaces. Furthermore, comparing the infectious samples of 3-log on the PFM and HFM, there was no dramatic difference in the Ct values, indicating that the PTI did not seriously damage the generic information of the H1N1pdm09 virus. Also, we performed simple RNA extraction-free qRT-PCR suggested by I. Smyrlaki et al*.*^11^ (Fig. S10). Without RNA extraction process, the Ct valuses of 3-log reference stock and 3-log on HFM with PTI were averagely 29.46 and 22.74, respectively. For both cases of 3-log H1N1pdm09 virus with and without the RNA extraction process, the Ct values were almost same, indicating that the RNA of H1N1pdm09 virus was naturally extracted by the PTI.

### Suggested PTI model of the H1N1pdm09 virus

The results of the TCID_50_ assay and crystal violet staining showed that the H1N1pdm09 virus lost its ability to infect mammalian cells under PTI. And, the Ct values of each infectious sample showed that the heat generated by the photothermal effect did not cause significant changes in genetic information, so that the M-gene of the H1N1pdm09 virus could be easily detected by qRT-PCR. Moreover, we confirmed that the genetic information of H1N1pdm09 virus was easily obtained via the RNA extraction-free RT-PCR.

**Figure 8:**
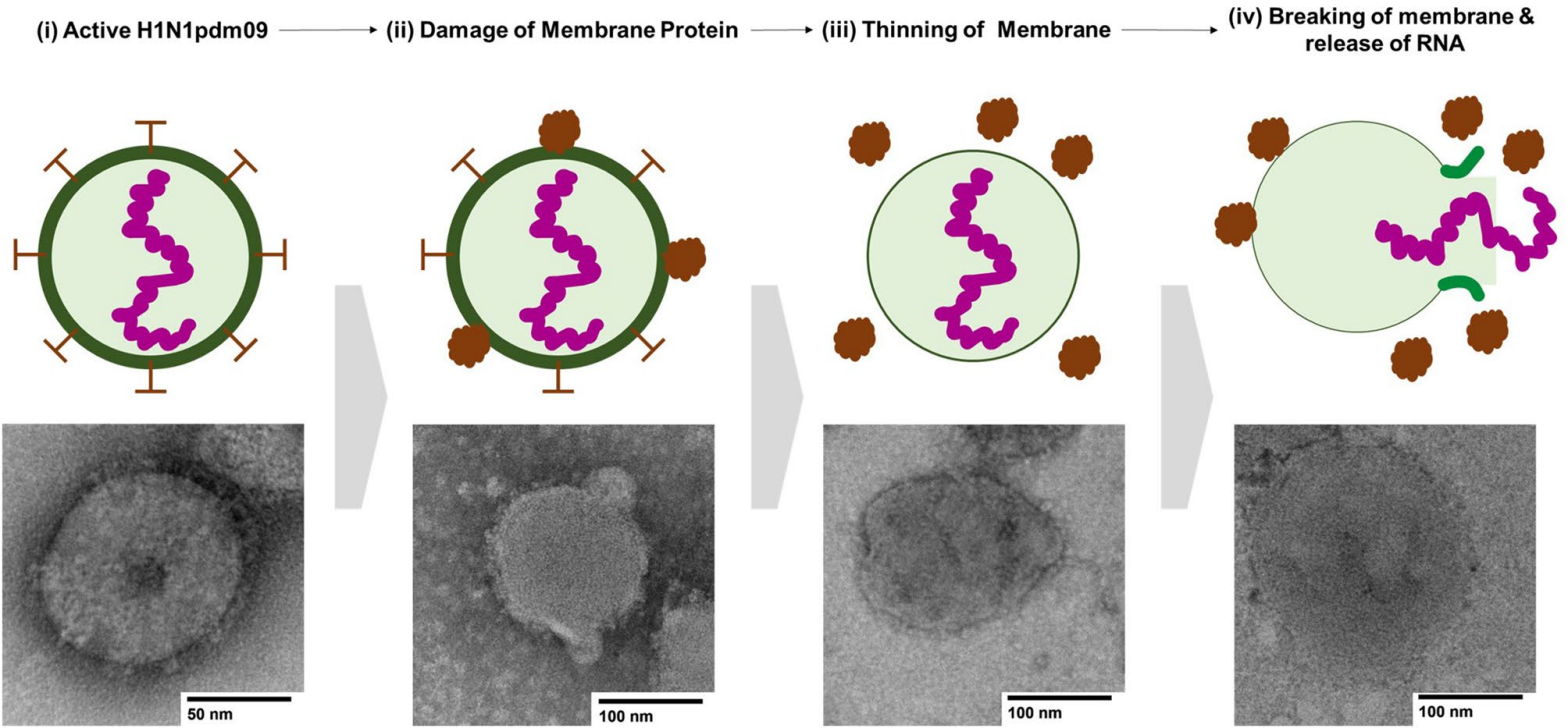
Suggested photothermal inactivation mechanism of the pandemic H1N1pdm09 virus.

In order to figure out PTI mechanism of H1N1pdm09 virus, we investigated the structural changes of H1N1pdm09 virus under the PTI via transmission electron microscopy (TEM). Figure 8 describes the suggested PTI model of the H1N1pdm09 virus. In this work, the HFM coated with the plasmonic Au NPs could absorb 560 nm LED light and immediately convert light energy into thermal energy, resulting in an increase in the surface temperature up to approximately 60 °C at RT. This range of surface temperatures could damage or kill the virus via thermal attack, leading to damage of membrane protein, the breakage or phase change of phospholipid bilayers over the transition temperature *T*_*m*_, which has an important effect on the structure of the biological membrane, including cells, bacteria, and viruses^45^. In addition, as a similar inactivation process to PTI, Korneev et al. suggested that photodynamic inactivation treatment of a virus induces the removal or damage of viral surface glycoprotein, leading to non-infectious viral particles^46^. Therefore, we expect that PTI could firstly destroy the viral membrane proteins via thermal attack as shown in TEM images of Fig. 8. Secondly, the continuous thermal attack can give rise to the thinner lipid bilayer of H1N1pmd09 virus. Eventually, the lipid bilayer of H1N1pdm09 virus is burst and the RNA of H1N1pdm09 virus is extracted outside the lipid bilayer of H1N1pdm09 virus.

In addition, Yap et al. introduced an analytical framework to understand the effects of temperature fluctuations on virus lifetime. This previous work suggested that the magnitude of temperature fluctuation become more significant than a steady-state mean temperature in predicting virus lifetime^47^. In the future, it would be valuable to investigate the structural changes of viral particles under thermal treatment and the effect of temperature fluctuation induced by the photothermal effect in detail.

## Conclusions

In summary, we fabricated an HFM conventionally spray-coated with plasmonic Au NPs to eliminate the infectivity of the corona-shaped H1N1pdm09 virus. The photothermal effect was significantly and immediately induced in the HFM under visible LED light irradiation, resulting in a rapid increase in the surface temperature to an average of 60 °C at RT. With this HFM, for the 3-log and the 4-log virus stock cases, the reduction rates of the virus titer reached > 99.9% in just 10 min. Moreover, the infectivity of the SARS-CoV-2 virus could be reduced by 99% using s-HFM as an alternative candidate for the HFM. However, at a higher concentration of 5-log of the virus stock, reduction rates of 97% and 99.7% were obtained after 10 min and 1 h of the photothermal treatment, respectively. In addition, the results of qRT-PCR showed that the M-gene of all infectious samples remained on the surface of the HFM without thermal damage. These results show that the PTI driven by the L-SPR of the plasmonic Au NPs could immediately and effectively inactivate the H1N1pdm09 virus. Furthermore, we can expect that viral inactivation originates from the denaturation of membrane proteins or the fragmentation of viral particles. As a result, this work provides an effective PTI method, which could be another candidate for reducing viral transmission in the field of air quality control in conjunction with the capture of viral particles by the filter materials and for extracting genetic information from photothermally inactivated viruses for qRT-PCR.

## Materials and methods

### Preparation of Au NPs

Plasmonic Au NPs were synthesized using a seed mediated method, as described elsewhere^48,49^. In a typical procedure, 50 mL of an aqueous gold chloride (HAuCl_4_; 2.0 × 10^–4^ M) solution is heated to its boiling point in a flask followed by the rapid addition of 0.5 mL of a sodium citrate (NaC_6_H_5_H_5_O_7_; 38.8 mM) solution. The color of the solution turns blue, finally changing to red after approximately 150 s, after which the solution is cooled to RT. The total reaction time is approximately 15 min.

### Viruses and cell samples

The H1N1pdm09 virus has been used to evaluate the PTI of viral particles^50^. We generated a recombinant pandemic H1N1pdm09 virus using a reverse genetic system, which was kindly provided by Dr. R. J. Webby (St. Jude Children’s Research Hospital, TN, USA). MDCK cells (CRL-2936) were obtained from the American Type Culture Collection (Manassas, VA, USA), and used as the infected host cells^51^. All virus experiments were conducted at the Korea Research Institute of Bioscience and Biotechnology (KRIBB, Daejeon, Repuplic of Korea) and were approved by and conducted in accordance with the guidelines of the Institutional Biosafety Committee (IBC, approval number KRIBB-IBC-20200213) of the KRIBB. In addition, for the inactivation test of SARS-CoV-2, the pathogen resources (NCCP43326) for this study were provided by the National Culture Collection for Pathogens.

### Numerical simulation of photothermal effect

The photothermal effect and temperature distributions were simulated using Ansys Fluent software (Ansys Inc., Pennsylvania, USA). Here, the material properties of Au NPs, such as the absorption cross-section and thermal conductivity, can be found in the literatures^52–54^. For the simulation of the photothermal effect and temperature distributions, we assumed that (1) The system was in a steady state; (2) The incident optical power of the light source was constant; and (3) There was no convective flow. We also focused on the absorption of light on the surface of the Au NPs in the scope of this work.

### Fabrication and characterization of HFM

For the PTI of H1N1pdm09 virus, we first prepared the HFM based on HEPA filter material purchased from Korea Coen, consisting of PET as a supporting layer and PP as a filtration layer. The as-synthesized Au NPs were conventionally spray-coated onto the surface of the supporting layer, which was then dried with nitrogen gas, and then baked in an oven for 2 h at 50 °C to evaporate excess solvent remaining on the surface of the HFM (Fig. S11). In addition, s-HFM was fabricated by conventional semiconducting process of sputtering evaporation. The base and deposition pressures of the chamber were 1 × 10^–5^ and 2.0 × 10^–3^ Torr, respectively. The deposition rate of Au and TiO_2_ were 10 nm/min at 40 W and 3.5 nm/min at 100 W, respectively. The target thickness of Au/TiO_2_ was 20 nm/200 nm.

After fabricating the HFM, infrared spectra of the HFM were obtained using a FTIR spectrometer (Nicolet iS50, Thermo Fisher Scientific Instrument, USA) in the range of 400–4000 cm^−1^ with a resolution of 2 cm^−1^. X-ray diffraction (XRD) results were obtained using an X-ray diffractometer (SmartLab, Rigaku, Japan) with Cu-Kα radiation operated at 40 kV and 30 mA in the 5° to 90° range of 2θ with an internal angle of 0.2°. The size and morphology of the Au NPs and filter fibers were characterized by scanning electron microscopy (SEM, JSM6500F, JEOL, Japan) and transmission electron microscopy (TEM, Tecnai G2 F30 S-TWIN, FEI Company, USA). Moreover, to measure the surface temperature of the HFM due to the photothermal effect, 530 and 560 nm LEDs (LED, SW530A(500mW class) and SW560A(800mW class), ORCA Science, USA) were used as light sources. Under irradiation by the light sources, the surface temperature was recorded using a thermal imaging camera (TIC, PI640, Optrics GmBH, Germany) at room temperature (RT, 20–25 °C).

### Determination of virus titer (or activity)

To determine virus titer (or activity), we used the TCID_50_ assay and crystal violet staining method, which relies on the morphological change of the host cells after being infected by the H1N1pdm09 virus^55,56^. The H1N1pdm09 virus titer was examined by injecting 100 μL of tenfold dilutions of infectious samples into wells seeded with MDCK cells and then calculating the TCID_50_ using the method suggested by Reed and Muench^57^ (More details in Supplementary Information).

### Detection of viral gene via qRT-PCR

We also conducted qRT-PCR to detect the matrix (M)-gene of the H1N1pdm09 virus using the gene-specific primer and hydrolysis probe set used in the qRT-PCR, as shown in Table 1. The hydrolysis probe was labelled with carboxyfluorescein (FAM) reporter dye at the 5’ end and with 6-carboxyltetramethylrhodamine (TAMRA) quencher dye at the 3’ end. The qRT-PCR, for detecting the M-gene of the H1N1pdm09 virus, was performed using a SenisiFAST Probe No-ROX One-Step KIT (BIOLINE, Taunton, MA, USA) (More details in Supplementary Information).

## Supplementary Information

Supplementary Information 1.

Supplementary Video 1.

Supplementary Video 2.
