# Supplementary material for: Photothermal inactivation of universal viral particles by localized surface plasmon resonance mediated heating filter membrane
Source: Sci Rep. 2022 Feb 2;12:1724. doi: 10.1038/s41598-022-05738-2 (PMC8810778; doi:10.1038/s41598-022-05738-2)
Supplement: Supplementary file 1 — Supplementary Information 1. [file 41598_2022_5738_MOESM1_ESM.docx]

**Supporting Information**

**Photothermal Inactivation of Universal Viral Particles by Localized Surface Plasmon Resonance Mediated Heating Filter Membrane**

Seunghwan Yoo^1,2,#^, Sun-Woo Yoon^3,4,#^, Woo-Nam Jung^5,6^, Moon Hyun Chung^1,2^, Hyunjun Kim^1^, Hagkeun Jeong^7^, and Kyung-Hwa Yoo^2,*^

^1^ Energy ICT Convergence Research Department, Energy Efficiency Research Division, Korea Institute of Energy Research, 152 Gajeong-ro, Yuseong-gu, Daejeon 34129, Republic of Korea.

^2^ Department of Physics, Yonsei University, 50 Yonsei-ro, Seodaemun-gu, Seoul 03722, Republic of Korea.

3 Biotechnology Research Center, Korea Research Institute of Bioscience and Biotechnology, 125 Gwahak-ro, Yuseong-gu, Daejeon 34141, Republic of Korea.

4 University of Science and Technology, 217 Gajeong-ro, Yuseong-gu, Daejeon 34114, Republic of Korea.

^5^ Advanced Combustion Power Lab., Energy Efficiency Research Division, Korea Institute of Energy Research, 152, Gajeong-ro, Yuseong-gu, Daejeon 34129, Republic of Korea.

^6^ Department of Mechnical Engineering, Korea advanced Institute of Science and Technology, 291 Daehak-ro, Yuseong-gu, Deajeon 34141, Republic of Korea.

^7^ Energy Efficiency Research Division, Korea Institute of Energy Research, 152, Gajeong-ro, Yuseong-gu, Daejeon 34129, Republic of Korea.

* Corresponding author. E-mail: khyoo@yonsei.ac.kr

# Equally contributed.


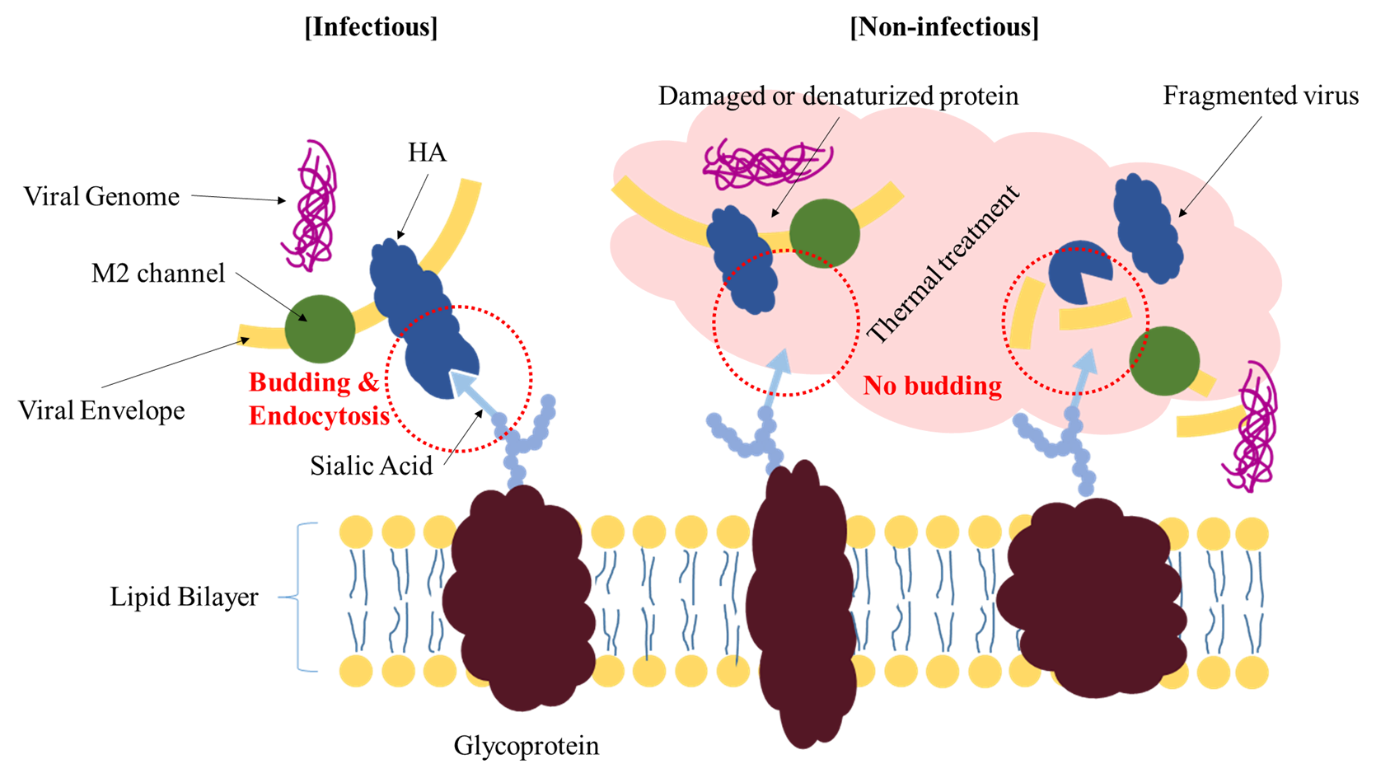


**Fig. S1**. Schematic illustration of the initial stage of virus infection and possible mechanism of the inactivation of the virus using photothermal treatment. (Infectious virus) hemagglutinin (HA) of the H1N1pdm09 virus buds to the sialic acid (DA) of glycoprotein embedded in the lipid bilayer of host cells, including endocytosis. (Non-infectious virus) Heat generated by the photothermal effect can damage or deform the membrane proteins or its component for inhibiting buds and endocytosis.


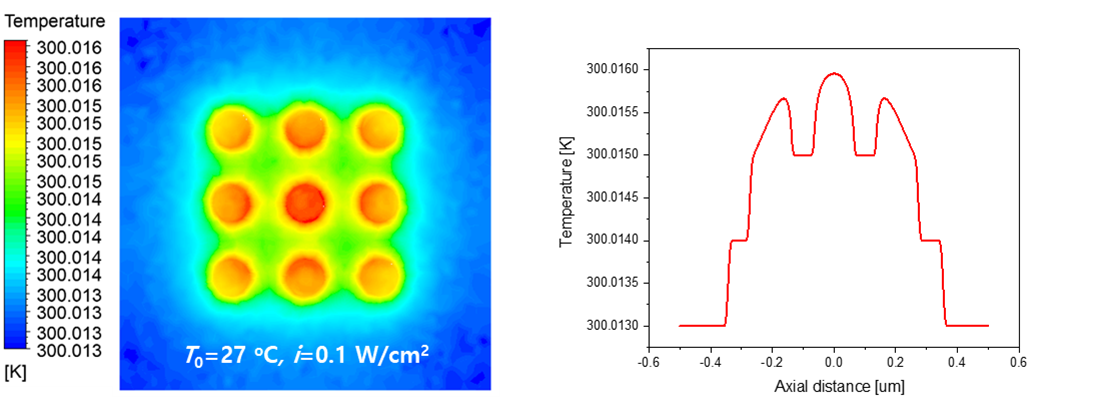


**Fig. S2** Simulation results of 3x3 Au NPs array under 100 mW/cm^2^. (Left) Contour image of photothermal effect generated by 3x3 Au NPs array. (Right) Cross-sectional temperature distribution of photothermal effect.


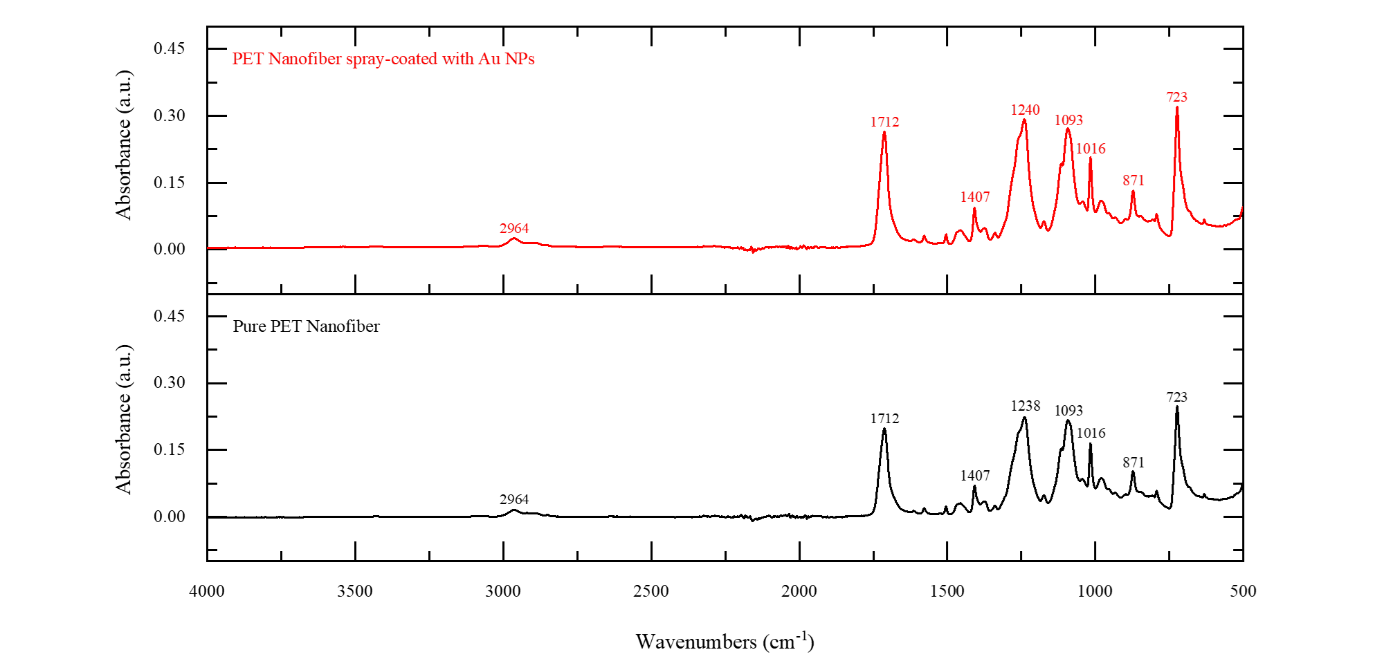


**Fig. S3** FTIR spectrum of the HFP and the PFP. The peak at 1712 cm^-1^ displays the C-O of ester groups, and the C-H out-of-plane deformation of two carbonyl groups on the aromatic ring depicts at 723 cm^-1^ [1]. The peaks at 1407 cm^-1^ and 1240 cm^-1^ represents the -H-C-H- (or -CH2-) deformation band and C(O)-O stretching of ester groups, respectively [2]. The symmetric aliphatic C-H stretching vibration was shown in 2964 cm^-1^ [2].

**Fig. S4** Increases of measured surface temperature of the HFP with respect to the optical power density of incident light under the illumination of 560 nm LED. The slop of increases is approximately 0.35 ℃/mW.


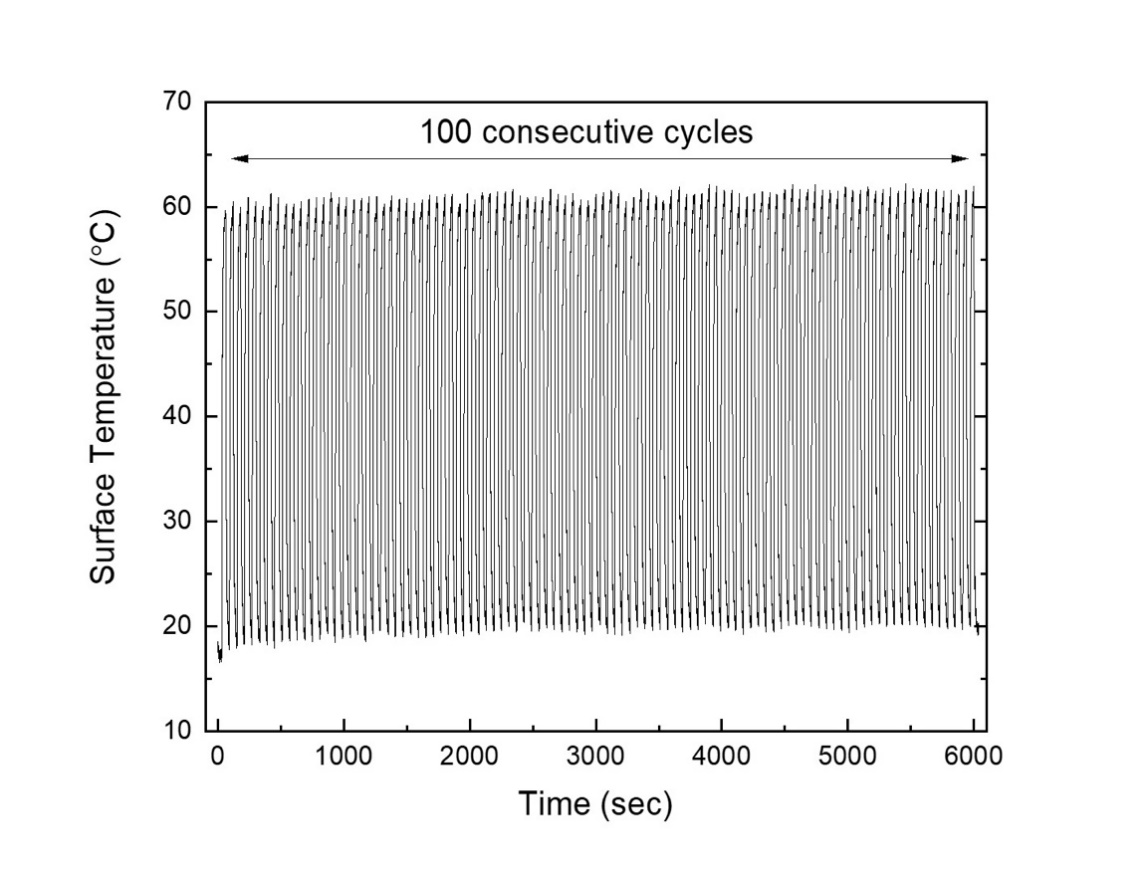


**Fig. S5** 100 consecutive cycles of measured surface temperature under 100 mW/cm^2^.


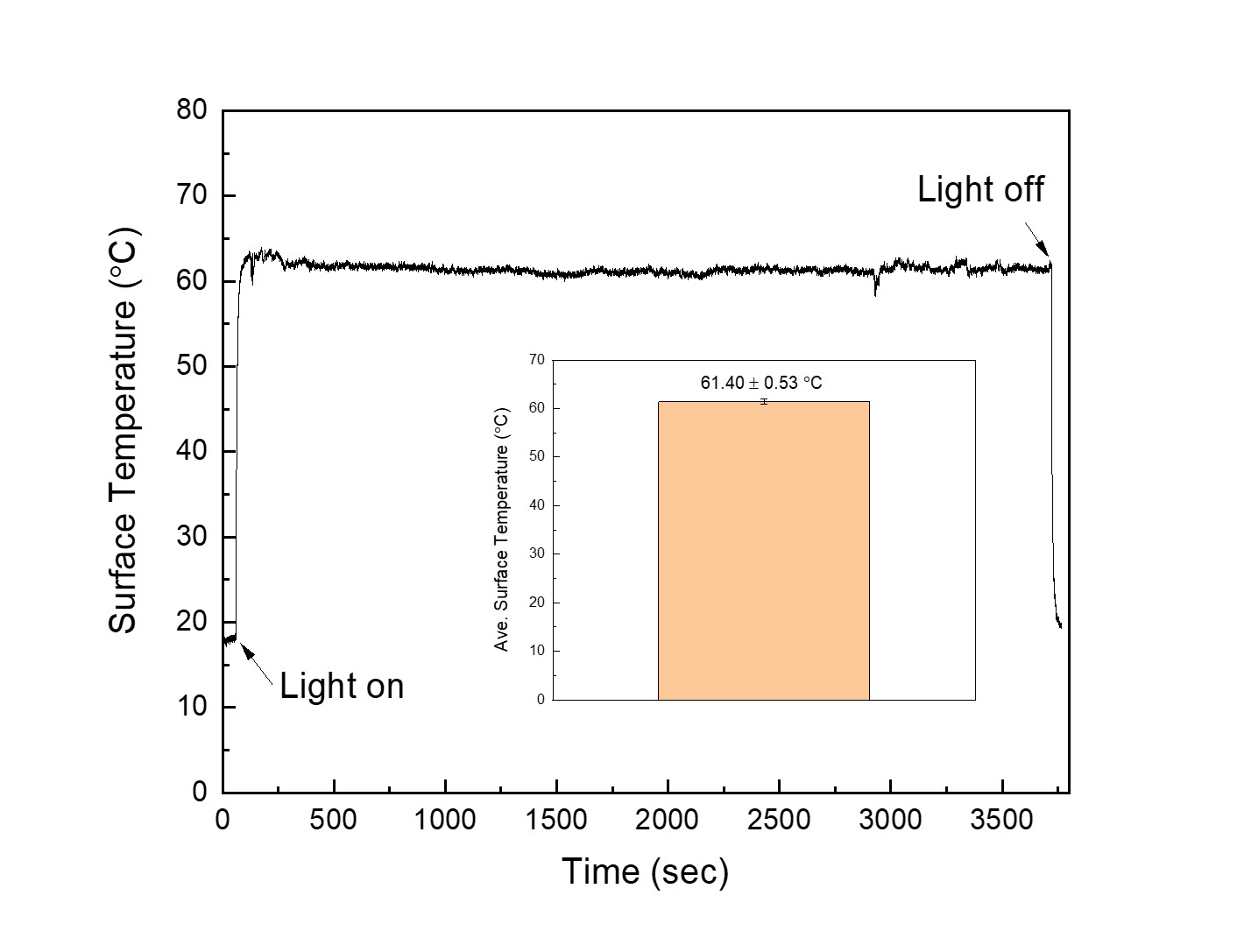


**Fig. S6** Monitoring the surface temperature fluctuation under continuous exposure of 560 nm LED light irradiation for 1 hour.


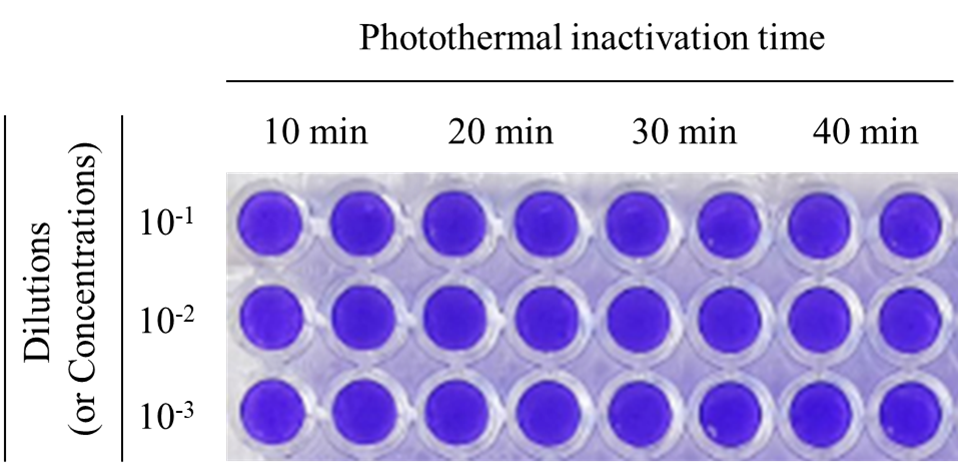


**Fig. S7** Photographs of the crystal violet staining of MDCK cells with respect to the irradiation time of photothermal inactivation (10, 20, 30 and 40 min).


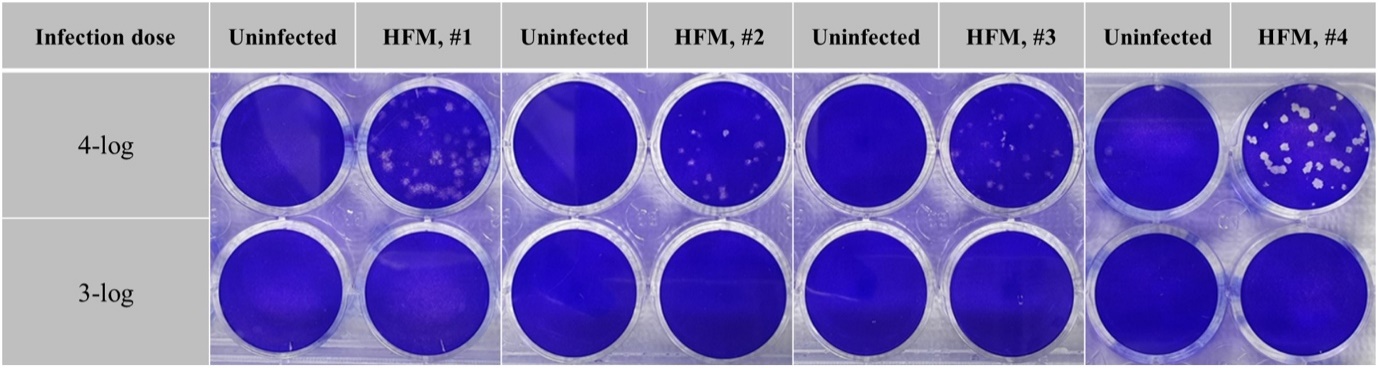


**Fig. S8** Photographs of plaque assays of SARS-CoV-2 virus inactivated by the photothermal effect of HFM.


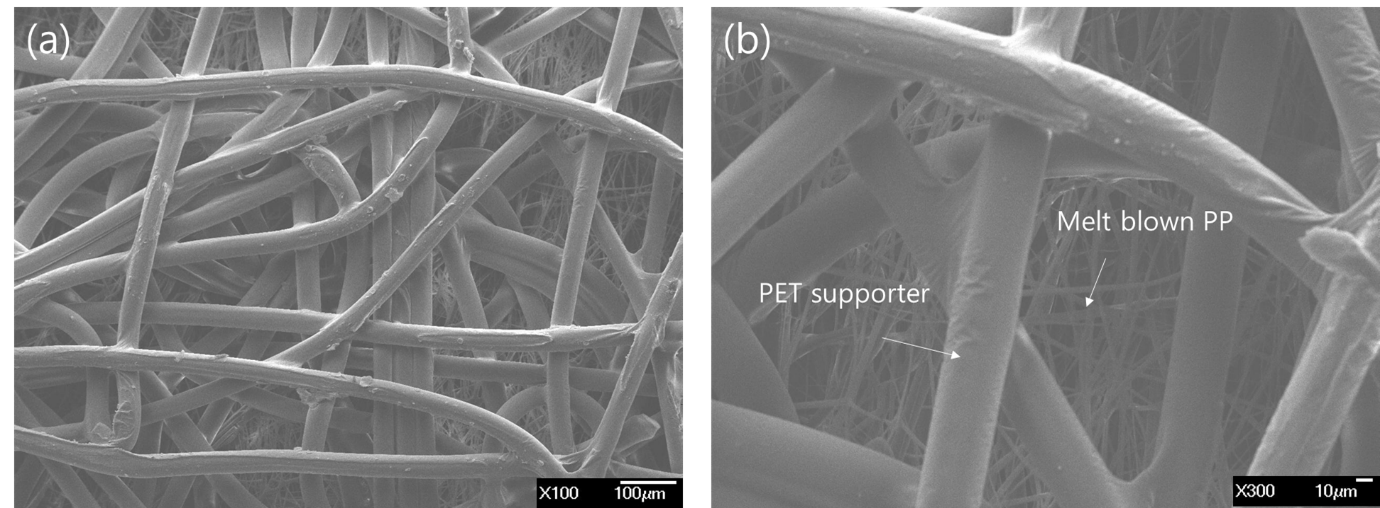


**Fig. S9** SEM images of s-HFM.


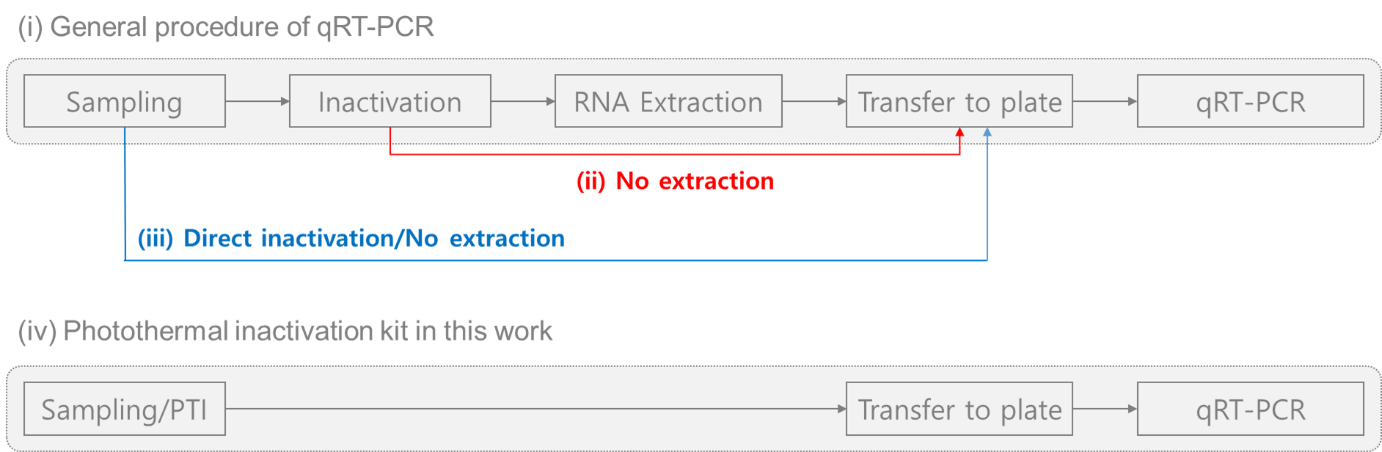


**Fig. S10** Flow diagram of general procedure of qRT-PCR and reduced procedure in this work [3].


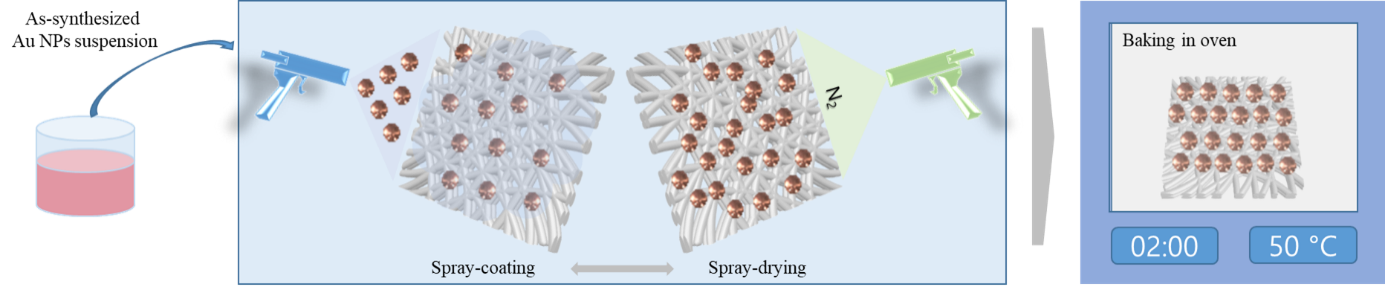


**Fig. S11** Simple fabrication process of the heating filter paper.

**Determination of virus titer (or activity)**

First, the MDCK cells (10^3^ cells/µL) were seeded in a growth medium in each well of a 96-well plate and incubated for 24 h at 37 °C in a 5% CO_2_ incubator. Simultaneously, we prepared 10-fold serial dilutions of the infectious viral samples in Dulbecco’s modified Eagle’s medium (DMEM) and washed the plate with 1× phosphate-buffered saline (PBS) to remove excess culture medium from each well. Each infectious viral sample was derived from the HFP, which was treated under the photothermal effect.

After preparing two kinds of experimental materials—that is, MDCK cells and viral samples—we transferred 100 µL of the diluted viral samples to the MDCK-seeded well plate using a multi-channel pipette, before removing the unbound samples and the cells by washing with a PBS solution after incubation for 1 h at 37 °C in a 5% CO_2_ incubator. To confirm the cytopathic effect (CPE) of MDCK cells under an inverted microscope, 200 µL of infection media was added to each well, the MDCK cells with the viral materials being incubated at 37 °C for 72 h in a 5% CO_2_ incubator. Finally, we calculated the TCID_50_ of each sample using eq. (A1):

$\log_{10} TCID_{50}=d_{h}+\frac{d}{2}-d \sum P_{i}$ (A1)

where dh is the logarithm of the highest dilution that shows 100 % CPE, d is the logarithm of the dilution ratio, and Pi is the positive response to dilution i.

After counting the number of wells with or without CPE, we removed the culture medium and rinsed each well with 100 µL of PBS. For fixation of the cells, 100 µL of 10% formaldehyde solution in PBS was added to each well, incubated for 10 min at RT and rinsed with 100 µL of PBS. Finally, 100 µL of crystal violet solution was added to each well and the cells were incubated for 10 min at RT. After discarding the crystal violet solution and rinsing with PBS, the well plate was dried, and the number of wells with or without CPE was counted using the naked eye.

**Determination of virus titer (or activity)**

The total volume of qRT-PCR components was 20 µL, consisting of 4 µL of template, 1 µL each of forward and reverse primer (M+25 and M-124), 1 µL of M+64 probe, 0.2 µL of reverse transcriptase, and 0.4 µL of RiboSafe RNase inhibitor. Distilled water was added, to a total volume of 20 µL.

With the qRT-PCR components, qRT-PCR was performed first with 1 cycle of reverse transcription at 45 °C for 10 min followed by 95 °C for 2 min. Moreover, 40 cycles of PCR were performed at 95 °C for 5 s and 60 °C for 2 s. The thermocycling process was driven by a LightCycler 96 System (Roche, Basel, Switzerland), and positive results were obtained based on an analysis of the fluorescent curves originating from each probe within 40 cycles.

**Reference**

[1] Das, O., Loho, T. A., Cepezza, A. J., Lemrhari, I. & Hedenqvist, M. S. A Novel way of adgering PET onto Protein (Wheat Gluten) plastics to impart water resistance. *coatings* **8**(11), 388 (2018).

[2] Paszkiewicz, S. et al. Synthesis and characterization of poly(ethylene terephthalate-co-1,4-cyclohexanedimethylene terephtlatate)-block-poly(tetramethylene oxide) copolymers. *RSC Advances* **7**, 41745-41754 (2017).

[3] Smyrlaki, I. et al. Massive and rapid COVID-19 testing is feasible by extraction-free SARS-CoV-2 RT-PCR. *Nature Comm.* **11**, 4812 (2020).
